# Supplementary material for: Causal Relationship between Aging and Anorexia Nervosa: A White-Matter-Microstructure-Mediated Mendelian Randomization Analysis
Source: Biomedicines. 2024 Aug 16;12(8):1874. doi: 10.3390/biomedicines12081874 (PMC11351342; doi:10.3390/biomedicines12081874)
Supplement: Supplementary file 1 [file biomedicines-12-01874-s001.zip › Supplementary Figure/Figure S8/Leaveoneout plot of MR analysis for TL to ubm-b-1875.pdf]

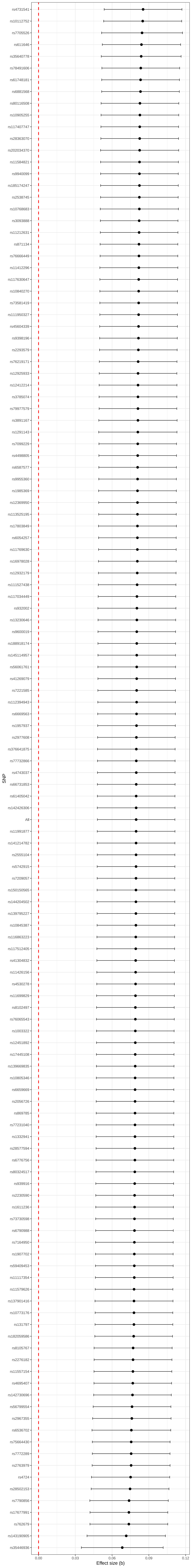
rs4731541
rs10112752
rs7705526
rs611646
rs35640778
rs78491606
rs61748181
rs6881568
rs80116508
rs10905255
rs117407747
rs28363070
rs202034370
rs11584821
rs9940099
rs185174247
rs2538745
rs10768683
rs3093888
rs11212631
rs871134
rs76666449
rs11412296
rs117630647
rs10840270
rs73581419
rs111950327
rs45604339
rs9398196
rs2293579
rs76219171
rs12925933
rs12412214
rs3785074
rs79977579
rs3891167
rs1291143
rs7099229
rs4498805
rs6587577
rs9955360
rs1985369
rs12369950
rs113525195
rs17803849
rs6054257
rs11769630
rs16978028
rs12932179
rs111527438
rs117034449
rs932002
rs13230646
rs9600019
rs188918174
rs145114957
rs56061761
rs41269079
rs7221585
rs112394943
rs6669563
rs1957937
rs2977608
rs376641875
rs77732866
rs4743037
rs66731853
rs61405042
rs142426306
All
rs11991877
rs141214782
rs2555104
rs5742915
rs7209057
rs150150565
rs144204502
rs139795227
rs10845387
rs116863223
rs117512405
rs41304832
rs11426156
rs4530278
rs11699829
rs8102497
rs76065543
rs1003322
rs12451892
rs17445108
rs139669835
rs10805346
rs6659669
rs2056726
rs869785
rs77231040
rs1332941
rs28577594
rs6776756
rs80324517
rs939916
rs2230590
rs1611236
rs73730598
rs6790988
rs7164950
rs1907702
rs59409453
rs11117354
rs11579626
rs137901416
rs10773176
rs131797
rs182059586
rs8105767
rs2276182
rs11557154
rs4695407
rs142730696
rs56799554
rs2967355
rs6536702
rs75664430
rs7772289
rs2763979
rs4724
rs28502153
rs7790856
rs17677991
rs762679
rs143190905
rs35446936
SNP
0.00
0.03
0.06
0.09
0.12
Effect size (b)
